# Endoscopic Screening and Surveillance of Gastrointestinal Cancer

**DOI:** 10.7759/cureus.79274

**Published:** 2025-02-19

**Authors:** Emilija Barauskaite, Andrius Raciunas, Rolandas Vaicekauskas

**Affiliations:** 1 Department of Family Medicine Center, Vilnius University Hospital Santaros Clinics, Vilnius, LTU; 2 Department of Gastroenterology, Nephrourology, and Surgery, Vilnius University Hospital Santaros Clinics, Vilnius, LTU

**Keywords:** cancer, endoscopic, gastrointestinal, screening, surveillance

## Abstract

Gastrointestinal (GI) cancer is a major health concern, contributing significantly to mortality rates in many regions, including Europe. It affects millions of people worldwide and leads to hundreds of thousands of deaths each year. Early detection and treatment through endoscopic methods play a vital role, providing less invasive and more affordable options compared to traditional surgical procedures. Targeted screening is vital for conditions such as Barrett's esophagus (BE), esophageal adenocarcinoma (EAC), gastric cancer (GC), ampullary carcinoma (AC), and colorectal cancer (CRC), particularly in high-risk populations. Endoscopic surveillance significantly reduces cancer incidence and improves survival rates, highlighting the importance of continuous advancements and updated guidelines to enhance screening efficacy and patient outcomes.

## Introduction and background

Across the European Union, cancer is the leading cause of death among individuals under 65 years, surpassing all other diseases [1]. Gastrointestinal (GI) cancer demonstrates significant global variability in its occurrence. It holds the third position in terms of incidence among men and the second among women globally, with an estimated total of more than 1.8 million cases diagnosed in 2018 [2]. In Europe, around 600,000 cases of GI cancer are diagnosed, leading to approximately 360,000 associated deaths each year [3].

Endoscopic procedures for the GI tract are specialized techniques crucial in early cancer detection and removing precancerous growths. Endoscopic procedures, despite challenges like limited access to certain areas, risks of bleeding or perforation, and difficulty in assessing tumor depth, have proven highly effective. They may not always detect microscopic cancer or treat advanced stages, but they significantly reduce cancer incidence and improve five-year survival rates for patients with early-stage disease. Patients often prefer these minimally invasive endoscopic procedures due to their less invasive nature and lower associated costs than traditional surgical interventions. Furthermore, endoscopic and histological examinations of precancerous conditions allow for a precise assessment of individual patient risk. This enables the tailoring of surveillance protocols, ensuring that high-risk individuals receive the most appropriate and beneficial follow-up care [4].

## Review

Methodology

This review was conducted through a comprehensive literature search and analysis of current guidelines and studies related to gastrointestinal (GI) cancer screening and surveillance. A systematic search was performed using the PubMed database. Keywords used included “GI cancer”, “endoscopic screening”, “Barrett’s esophagus”, “gastric cancer”, “ampullary carcinoma”, and “colorectal cancer”. Search results were filtered to include peer-reviewed articles, systematic reviews, meta-analyses, and clinical guidelines published from 2015 to 2024. Articles were included if they focused on endoscopic screening and surveillance guidelines for GI cancers, including Barrett’s esophagus, gastric cancer, ampullary carcinoma, and colorectal cancer. Studies focusing on non-endoscopic methods or experimental techniques without clinical application were excluded. Recommendations from leading professional societies, including the European Society of Gastrointestinal Endoscopy (ESGE), American Gastroenterological Association (AGA), American Society of Gastrointestinal Endoscopy (ASGE), and British Society of Gastroenterology (BSG) were reviewed and compared. Information regarding screening protocols, risk factors, and surveillance intervals was extracted and organized into comparative tables. Data synthesis focused on identifying patterns and differences in guidelines, emphasizing high-risk populations and surveillance intervals based on risk factors. This methodology ensured a comprehensive, evidence-based analysis of current screening and surveillance strategies for GI cancers, supporting the development of clear and practical recommendations.

Barrett's esophagus and esophageal adenocarcinoma

Barrett's esophagus (BE) is a pathological condition characterized by replacing the normal squamous epithelium in the distal esophagus with metaplastic columnar epithelium. This metaplastic transformation is recognized as a premalignant condition, significantly increasing the risk of developing esophageal adenocarcinoma (EAC) [5]. EAC poses a significant global health burden. It ranks eighth in terms of incidence and sixth in terms of cancer-related mortality [6]. Although BE is uncommon in the general population, its prevalence rises to 5-15% in individuals with gastroesophageal reflux disease (GERD) and associated risk factors [7,8].

Screening for Barret's Esophagus and Esophageal Adenocarcinoma

**Figure 1:**
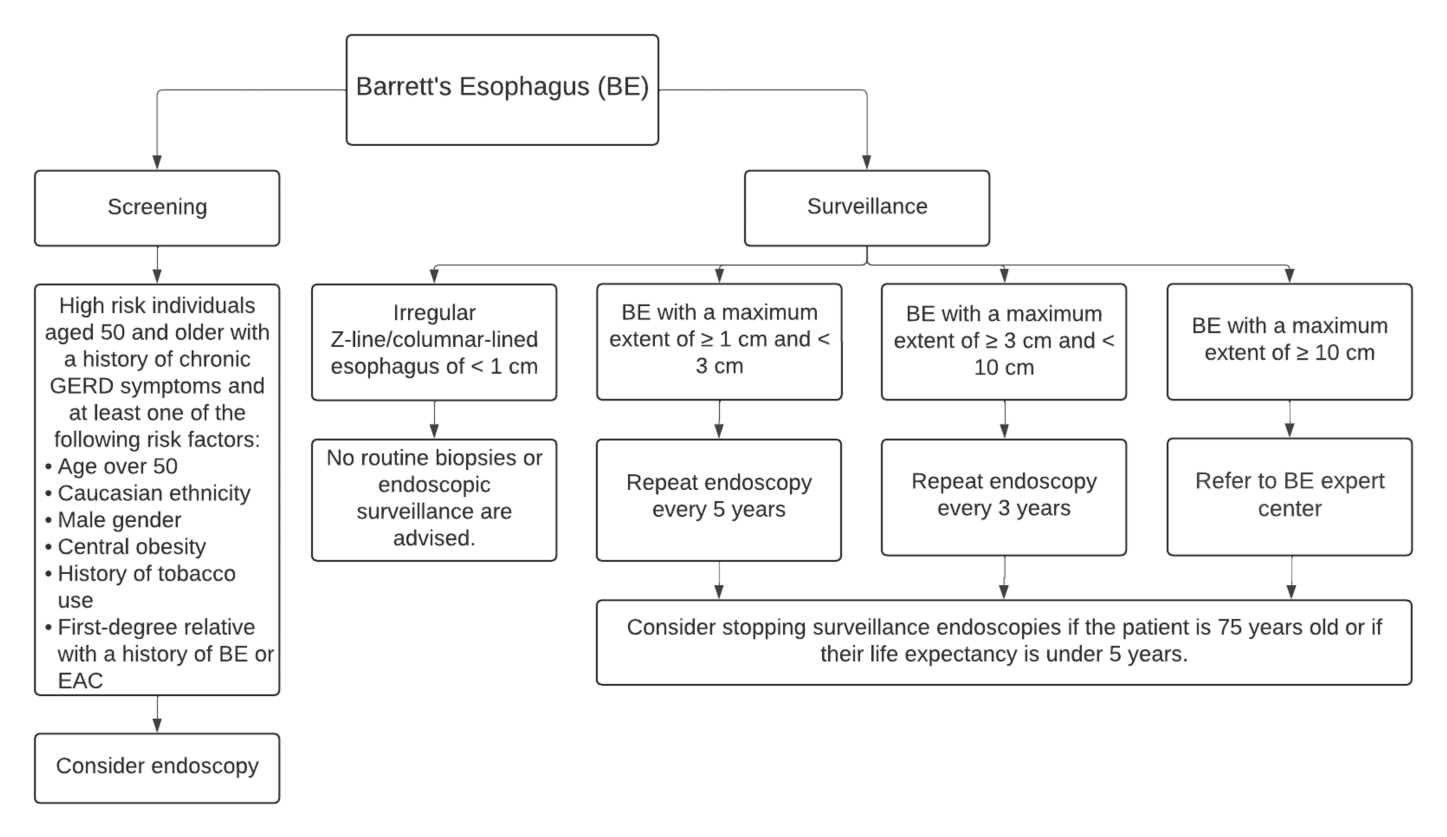
Screening and surveillance of Barrett's esophagus BE - Barrett's esophagus, EAC - esophageal adenocarcinoma, GERD - gastroesophageal reflux disease This table is an original creation by the authors.

Screening can reduce EAC-related mortality, but according to the European Society of Gastrointestinal Endoscopy (ESGE), it is not cost-effective for the general population due to the low prevalence (1-2%) and low annual progression risk (<1%) [5,9,10]. Screening is recommended in individuals with chronic GERD who also exhibit the following risk factors: age over 50 years, male sex, Caucasian ethnicity, central obesity, current or past tobacco use, and a first-degree relative who has been diagnosed with BE or EAC (Figure 1). The likelihood of developing BE rises by 1.2% with every additional risk factor [11]. Notably, around 50-60% of BE cases are found in individuals who do not exhibit GERD symptoms. According to the American Gastroenterological Association (AGA), screening can be considered for those with or without GERD if they possess at least three risk factors. This suggests that GERD might not be a critical criterion for BE screening [12].

The ESGE recommends BE screening in patients aged 50 years or older with chronic GERD and at least one additional risk factor [5,13], while the American Society of Gastrointestinal Endoscopy (ASGE) defines at-risk groups as those with a family history of BE/EAC or GERD plus one other risk factor [14]. The American College of Gastroenterology (ACG) provides more detailed criteria, recommending screening primarily for men with chronic GERD and two or more risk factors, while advising against routine screening in women unless multiple risk factors are present [10].

Surveillance of Barret's Esophagus and Esophageal Adenocarcinoma

Endoscopic surveillance is the gold standard for detecting dysplasia and neoplasia in patients with known BE. The frequency of surveillance depends on the length of BE and the degree of dysplasia. The ESGE recommends different surveillance intervals based on BE length (Figure 1). For BE extending 1 to 3 cm, surveillance should occur every five years. For BE extending 3 to 10 cm, the interval should be three years. Patients with BE extending 10 cm or more should be referred to a BE expert center for surveillance. No routine biopsies or endoscopic surveillance are advised for patients with an irregular Z-line/columnar-lined esophagus of less than 1 cm. Discontinuation of surveillance endoscopies should be considered if a patient has reached 75 years of age or if their life expectancy is less than five years [5].

Both the ACG and ESGE recommend endoscopic treatment for low-grade dysplasia, but if this is not carried out, they advise surveillance every six months. For high-grade dysplasia, endoscopic therapy is the preferred approach, with surveillance every three months if treatment is not feasible [5,10].

Gastric cancer

Gastric cancer (GC) is the world's fifth most prevalent cancer and the third leading cause of cancer-related deaths [15,16]. Around 90% of GC cases are linked to *Helicobacter pylori* (*H. pylori*) infection [17]. Recent studies indicate that eradicating *H. pylori* among healthy individuals can reduce the incidence of GC and mortality [18]. The 2020 Taipei global consensus confirmed that testing and treating all high-risk individuals (a family history of stomach cancer, pernicious anemia, tobacco smoking, high intake of meat, obesity, alcohol consumption) for *H. pylori* is warranted. It also suggested that mass screening and eradication should be considered in high-risk area populations [19]. Non-invasive tests like stool antigen, urea breath, and serological tests support a "test-and-treat" strategy for primary prevention in high-incidence areas [15]. Biomarkers like low serum pepsinogen have limited evidence for widespread screening [20].

Endoscopic Screening of Gastric Cancer

**Figure 2:**
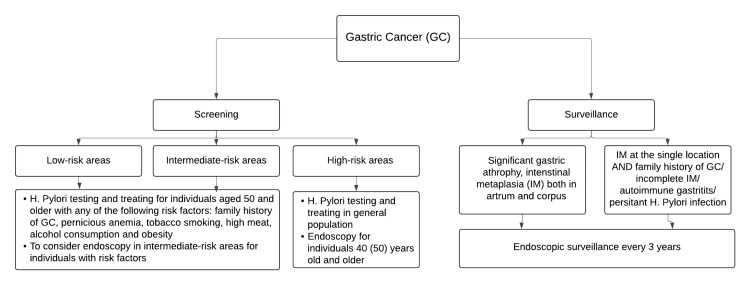
Screening and surveillance of gastric cancer GC - gastric cancer, IM - intenstinal metaplasia This table is an original creation by the authors.

There is insufficient evidence to support the efficacy of screening for GC in low-risk populations undergoing routine diagnostic procedures. In countries with intermediate GC incidence, it is recommended to consider endoscopy screening for only those with known risk factors for GC (older than 50 years, male sex, smoking, pernicious anemia, a family history of GC). Endoscopic screening should be considered in high-risk populations, especially individuals over 40 years of age (shown in Figure 2) [15,20].

Asian countries such as South Korea and Japan lead the mass population screening for GC. Their guidelines recommend endoscopic screening every two years for asymptomatic adults, with Japan targeting ages 50-75 and South Korea targeting ages 40-75 years. This recommendation is based on the estimated doubling time of gastric cancer [17,21].

In Europe, the British Society of Gastroenterology (BSG) advocates for endoscopic screening in individuals over 50 who have additional high-risk factors, including being male, smoking, having pernicious anemia, or having a family history of gastric cancer [20]. The Maastricht VI/Florence consensus also supports endoscopic screening with biopsies for asymptomatic individuals with a family history of gastric cancer starting at age 45 years or older [22]. Endoscopic GC screening is not recommended for adults over 75 years old due to a lack of evidence supporting its benefits beyond this age. *Surveillance of Gastric Cancer* The AGA and ASGE both recommend against routine surveillance for gastric intestinal metaplasia (GIM), but suggest considering it for high-risk individuals, such as those with a family history of gastric cancer [23]. In contrast, the ESGE provides more specific guidance. European guidelines state that patients with mild to moderate atrophy confined to the antrum do not need surveillance. Those with intestinal metaplasia (IM) at a single site have an elevated risk of GC, but routine surveillance is generally not recommended. However, for patients with IM at a single site who also have a family history of GC, incomplete IM, or persistent *H. pylori* infection, endoscopic surveillance with chromoendoscopy and targeted biopsies may be considered every three years. Patients with advanced atrophic gastritis affecting the antrum and corpus should undergo high-quality endoscopy every three years. If these patients also have a family history of GC, it further elevates the risk of gastric cancer; therefore, more frequent follow-up, like every one to two years, may be beneficial (Figure 2) [20,21,24].

The ESGE recommends endoscopic resection as the preferred treatment for both low-grade and high-grade gastric dysplasia to ensure complete removal and accurate histological assessment. If resection is not feasible for low-grade dysplasia, surveillance endoscopy every six to 12 months is advised, while for high-grade dysplasia, post-resection surveillance should occur every three months in the first year, then annually [24].

Ampullary carcinoma

Ampullary carcinoma (AC) is a rare cancer originating at the ampulla of Vater, where the bile and pancreatic ducts empty into the duodenum. Its location often causes early symptoms like jaundice [25,26]. *Screening of Ampullary Carcinoma* Routine screening is not established for the general population but is recommended for high-risk groups, including individuals with Familial Adenomatous Polyposis (FAP), Lynch syndrome, or chronic pancreatitis [25,26]. For those with FAP or genetic predispositions, biannual endoscopic surveillance starting in their 20s is advised to detect early adenomas or carcinoma. Tumor markers (e.g., CA19-9, CEA) lack sensitivity for early detection but aid prognosis and monitoring. Genetic testing and counseling are advised for hereditary conditions like FAP [25,26]. *Surveillance of Ampullary adenomas* Ampullary adenomas (AA) can progress to carcinoma. Adenomas <1 cm are monitored with endoscopy every 6-12 months, while those >1 cm should be removed endoscopically [25,26].

Colorectal cancer

**Figure 3:**
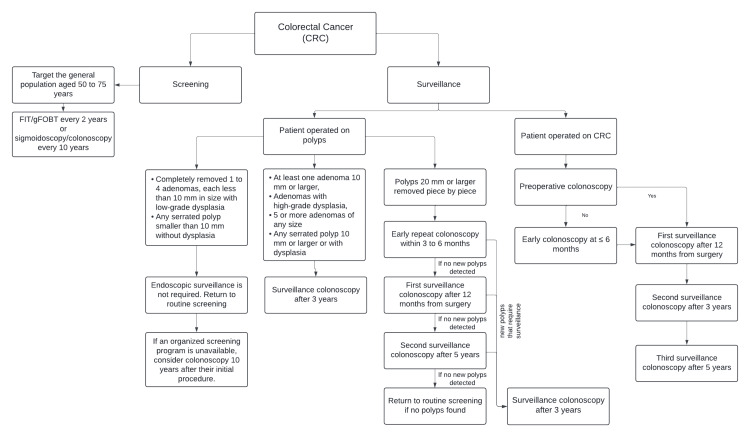
Screening and surveillance of colorectal cancer CRC - colorectal cancer, FIT - fecal immunochemical test, gFOBT - guaiac-based fecal occult blood test This table is an original creation by the authors.

Colorectal cancer (CRC) constitutes a considerable health challenge in the EU, ranking second in terms of cancer-related morbidity and mortality. In 2022, an estimated 539,000 new CRC cases and 248,000 CRC-related deaths were reported [27]. Established risk factors for CRC include age over 45 years, a family history of CRC, inflammatory bowel disease, obesity, diabetes, tobacco use, excessive alcohol consumption, excessive consumption of processed meat, and a sedentary lifestyle [28]. *Screening for Colorectal Cancer and Adenomatous Polyps* CRC is particularly well-suited for screening programs. This suitability is due to the presence of detectable precursor lesions, known as adenomatous polyps. The removal of these polyps significantly decreases both the incidence and prevalence of CRC, leading to a subsequent reduction in mortality [29,30]. Most screening programs target the general population aged 50 to 75 years. However, the age range may vary depending on resource availability (Figure 3). In the United States, the target age group was recently expanded to include individuals aged 45 and older due to an increased incidence of CRC among younger adults. This rising trend in CRC incidence has also been observed in Europe over the past 25 years [31-33].

In addition, the ACG guidelines recommend for individuals with one first-degree relative diagnosed with CRC or an advanced adenoma at age 60 or older, colonoscopy screening should begin at age 40 and be repeated every 10 years. If a single first-degree relative was diagnosed before age 60, or if two or more first-degree relatives have a history of CRC or advanced adenomas at any age, screening should begin at age 40, or 10 years younger than the youngest affected relative's diagnosis, whichever is earlier. In these higher-risk cases, colonoscopies should be repeated every 5 years [33].

Guaiac‐Based Faecal Occult Blood Tests (gFOBT) Versus Faecal Immunochemical Tests (FIT)

CRC programs in the European Union (EU) invite men and women for screening every 2 years with the gFOBT or FIT or every 10 years or longer with flexible sigmoidoscopy or total colonoscopy [34]. A gFOBT was effective but prone to false positives from diet and medications and false negatives from antioxidants. Unlike the older gFOBT, FIT requires fewer stool samples and uses antibodies specific to human hemoglobin, reducing interference from diet and medications and not requiring dietary restrictions [35-38]. Studies show that FIT demonstrates superior performance, with a sensitivity of 92.1% (95% CI: 86.9%-95.3%) and a specificity of 85.8% (95% CI: 78.3%-91.0%), compared to gFOBT, which has a sensitivity ranging from 31% to 79% and a specificity between 87% and 98% [39,40]. *Surveillance of Colorectal Cancer* ESGE recommends that patients undergoing surgery and appropriate oncological treatment for CRC receive post-surgery endoscopic surveillance. This includes a high-quality perioperative colonoscopy either before the CRC surgery or within six months following the procedure (Figure 3). A surveillance colonoscopy is strongly recommended one year after CRC surgery [41].

ESGE advises against an intensive endoscopic surveillance strategy, such as an annual colonoscopy, due to its lack of proven benefit. After the initial surveillance colonoscopy following CRC surgery, it is suggested that the second colonoscopy be done three years later and the third five years after. More frequent surveillance examinations may be considered if high-risk neoplastic lesions are detected [41]. Additionally, it is recommended to discontinue post-surgery endoscopic surveillance after the first surveillance colonoscopy at the age of 80 or sooner if comorbidities reduce the patient's life expectancy [41,42]. However, ACG recommends individualized screening decisions for patients over 75 years and should be based on life expectancy, risk factors, values, and preferences, using shared decision-making, while awaiting further research [33].

Surveillance of Adenomatous Polyps

There is a different approach to adenomatous polyps’ surveillance following endoscopic removal. Patients with 1 to 4 adenomas, each less than 10 mm in size with low-grade dysplasia, do not require endoscopic surveillance. Similarly, patients with any serrated polyp smaller than 10 mm without dysplasia should return to routine screening rather than undergo endoscopic surveillance. If an organized screening program is unavailable, these patients should have a repeat colonoscopy 10 years after their initial procedure [42].

Patients with more significant findings (one adenoma 10 mm or larger, adenomas with high-grade dysplasia, five or more adenomas of any size, or any serrated polyp 10 mm or larger or with dysplasia) should have a surveillance colonoscopy after three years [42,43]. A meta-analysis, providing moderate-quality evidence, revealed that advanced neoplasia was detected during follow-up in 3.6% of patients with low-risk findings and in 1.6% of patients without neoplasia [44]. In cases where polyps 20 mm or larger were removed piece by piece, an early repeat colonoscopy is recommended within three to six months to ensure complete removal [42].

Following this early repeat colonoscopy, the first surveillance colonoscopy should occur 12 months later to check for late recurrence. If the initial surveillance colonoscopy does not reveal any new polyps needing monitoring, a follow-up colonoscopy should be conducted five years later. Patients can be returned to routine screening if this second surveillance colonoscopy shows no polyps requiring further monitoring. However, if polyps requiring surveillance are detected at any of these examinations, subsequent colonoscopies should be conducted every three years [42].

*Hereditary Colorectal Cancer Syndromes* Hereditary syndromes (2-5% of CRC cases) include Lynch syndrome (non-polyposis) and polyposis syndromes [45]. Lynch syndrome, also known as hereditary non-polyposis CRC, is the most prevalent non-polyposis syndrome, contributing to approximately 2-4% of all CRC cases. Genetic counseling and individualized surveillance are recommended based on family history and genetic mutations [46]. For individuals with Lynch syndrome, annual colonoscopies may be recommended beginning between ages 20-25, or 10 years before the youngest age at which a family member was diagnosed with colon cancer, whichever comes first [46].

Familial Adenomatous Polyposis (FAP) is a disease caused by a dominant mutation in adenomatous polyposis coli gene [45-47]. Left untreated, FAP leads to the development of numerous polyps in the colon and rectum, often starting in adolescence. Without appropriate screening and management, nearly all individuals with classic FAP will develop colorectal cancer in a near-certainty of colorectal cancer, often by age 30-40 years [46].

For asymptomatic individuals with FAP, the ESGE recommends colonoscopy surveillance starting at ages 12-14 years, whereas the National Cancer Comprehensive Network guidelines advise annual sigmoidoscopy or colonoscopy for those with a confirmed APC gene mutation, beginning between ages 10 and 15 years [46,48].

Other Diseases Associated With Colorectal Cancer

Inflammatory bowel diseases (e.g., ulcerative colitis, Crohn's disease) increase CRC risk two to three times, especially with primary sclerosing cholangitis [49-52]. People who have a first-degree relative who has been diagnosed with CRC or an advanced adenoma are advised to have a colonoscopy every five to 10 years, starting at age 40-50 years or 10 years before the relative's age at diagnosis. Alternatively, fecal immunochemical testing every one to two years may be considered. Individuals with first-degree relatives who have non-advanced adenomas or a history of CRC in second-degree relatives should follow CRC screening guidelines [32,53].

## Conclusions

In summary, endoscopic screening and surveillance are vital for the early detection and management of GI cancer. These cancers, which include esophageal, gastric, duodenal, and colorectal types, pose significant global health challenges, especially in high-risk populations. Endoscopic techniques are highly effective in spotting precancerous conditions and early-stage cancers, leading to high survival rates and patient preference due to their minimally invasive approach. Specific guidelines and recommendations have been developed for the screening and monitoring of GI cancer, tailored to the risk profiles and demographics of different patient groups. These strategies are crucial for reducing the incidence and mortality of GI cancers. Continuous research and updates to these guidelines are necessary to improve the effectiveness and accessibility of these programs, ultimately enhancing patient outcomes and quality of life.
